# Editorial: The microbiome as a source of new enterprises and job creation

**DOI:** 10.1111/1751-7915.13026

**Published:** 2017-12-04

**Authors:** Jan‐Ulrich Kreft

**Affiliations:** ^1^ Institute of Microbiology and Infection and Centre for Computational Biology and School of Biosciences The University of Birmingham Birmingham B15 2TT UK

This editorial is part of a series that seeks to highlight the commercial potential of microbiome research. Such translation of scientific advances into jobs that improve the environment or human well‐being is an ethical imperative. It is also a scientifically selfish imperative as returning taxpayers' investments into science will also pay off for us scientists. Moreover, it is important scientifically because the ultimate test of scientific ideas and understanding is whether they can be put into practice.

It has long been known that germ‐free animals are poorly creatures, but it has taken until the explosion of research on the gut microbiota, mostly driven by the next‐generation sequencing revolution over the last decade, that we have begun to realize the profound effects that the microbes we contain have on us. Discussing these effects could fill a book, so a few examples have to suffice. The presence of a particular strain of *Enterobacter cloacae* can cause obesity by stimulating chronic inflammation (Zhao, 2013). Several Ruminococcaceae OTUs, as well as fibre intake and gut microbiota diversity, are linked to lower long‐term weight gain in a cohort of twins after adjusting for calorie intake (Menni *et al*., 2017). Microbes can be expected to have evolved to manipulate us into eating what *they* like (Alcock *et al*., 2014). We have to realize that to successfully treat a patient, we will often need to consider the patient's microbiota. Yet, we are far from understanding the multitude of interactions going on. The commonly used word ‘dysbiosis’ is an admission of our lack of causal understanding of diseases involving the microbiota.

Crucially, the microbiota is complex, the diet is complex, the host is complex and the interactions between members of the microbiota as well as between the microbiota and host are complex. I would like to argue that this poses a formidable scientific challenge that requires a massive effort with mathematical modelling at its core to overcome. Mathematical models on their own will not suffice, but without them, there is little hope for a rational engineering of the microbiota and investing into new products will be a gamble. Investing into staff and developing mathematical modelling platforms should pay off for biotech companies, as it should at least eliminate those product leads that have little chance of success in clinical trials.

For example, probiotics, despite considerable research efforts over decades, have not been the huge success that one would expect, given that the gut microbiota is implicated in many increasingly common diseases that the right probiotics should be able to cure. The limited success is partially due to a lack of mechanistic understanding that could be used in predictive mathematical models to select better (combinations of) probiotics and partially due to an inability of the typically used strains to colonize the gut. Having evolved in different environments, they are outcompeted by the indigenous microbes. We have been betting on the wrong probiotic horses. This is why beneficial effects, if found at all, have typically been found in underpowered studies that are likely to come up with false positives. One recent success, a large Indian study of a Lactobacillus strain specifically chosen for its ability to attach to epithelial cells, is a notable exception (Panigrahi *et al*., 2017).

Moreover, it is becoming more and more clear that increasing the diversity of the microbiota is beneficial for our health, implying that consuming only a few strains of probiotics is simply not enough. This is corroborated by the success of faecal microbiota transplants (FMT), which contain thousands of strains. Another reason for improving microbiota diversity is the increased protection against infections provided by a more diverse microbiota, which will contribute to reducing the use of antibiotics for treating animal and human disease and thereby help reduce antibiotic resistance.

There are several ways in which hiring mathematical modellers or cofunding external research could benefit probiotics companies. The research programme could start with identifying indigenous human gut microbes associated with health by comparing the gut community compositions of healthier humans with those who have various metabolic or gut issues while trying to remove confounding effects. Such efforts have moved on from the initial underpowered and overly generalizing studies that were focussed on phylum‐level comparisons towards more detailed analysis. Nevertheless, these studies already identified potential new probiotics, although it would be wise to stratify people by age, sex, diet and ethnicity, for example, as it is likely that the fate and benefits of probiotics depend on these factors (Menni *et al*., 2017). Alongside identifying probiotic candidates, one could determine positive and negative associations of these candidates with other candidates or other community members to combine individual candidates into cocktails of potentially health‐promoting microbes. Most studies to date take snapshots of the microbiota composition, sometimes with another snapshot at a later time, but rarely time series that are immensely valuable for understanding dynamical systems. For example, if time series are available, one can use generalized Lotka–Volterra models to infer the strength of positive and negative interactions between microbes by fitting such models to time series data (Stein *et al*., 2013). A great example of applying this approach is the identification of a relative of *Clostridium difficile* that inhibits *C. difficile* by converting a bile acid (Buffie *et al*., 2014). Another ignored aspect of the gut microbiota is its spatial organization, usually destroyed when extracting DNA, although spatial structure has been extensively shown to have profound effects on population dynamics and ecosystems (Hellweger *et al*., 2016). Notably, bottom‐up mathematical models can predict the self‐organization of microbes into spatial clusters and the effects these emergent structures can have on function (Hellweger *et al*., 2016).

The next step could be to reconstruct the genomes of these candidate probiotics from shotgun metagenomic sequences. This is not an easy task, but there has been great progress with binning of contigs based on co‐abundance or sequence signatures or a combination of both (Albertsen *et al*., 2013; Alneberg *et al*., 2014; Nielsen *et al*., 2014). Annotating these reconstructed genomes could indicate carbon and nitrogen utilization pathways and culturing conditions that should help to enrich these species and bring them into pure culture. This strategy facilitated the isolation of a Succinivibrionaceae strain that is dominant in the Tammar wallaby, of particular interest due to the reduced methane emission of wallabies compared with ruminants (Pope *et al*., 2011).

Moreover, the statistical associations and dynamic model inferred interactions between species could be integrated with mechanistic predictions of interactions based on genome‐scale metabolic models. Starting with reconstructed genomes or genomes obtained from pure cultures, one can build stoichiometric matrices (essentially lists of enzyme reactions potentially carried out by these species) and use flux balance approaches to predict metabolic phenotypes and which metabolites could be utilized and produced by each species, thus predicting metabolic interactions such as competition or cross‐feeding (Shoaie *et al*., 2015; Magnúsdóttir *et al*., 2017). Together, this information would be very useful in picking an optimal mix of probiotic microbes for a particular group of humans.

In addition to inferring and predicting interactions and metabolic properties, mathematical modelling can be hugely beneficial when used to reduce the complexity of a system, e.g. by lumping isolates or sequence OTUs with similar metabolism into functional groups and then checking whether this simplified system is sufficient to explain the key characteristics of the system one is interested in. For example, Kettle *et al*. (2014) compared a mathematical model with ten metabolic groups to data from an *in vitro* model inoculated with human gut microbiota. Their results suggest that ten functional groups are sufficient to describe the fermentative metabolism in the colon.

In contrast to probiotics, FMT has been a huge success (van Nood *et al*., 2013), likely due to the high diversity of transferred microbes compared with probiotics. However, it is only a question of time before the faecal microbiota transferred from a healthy donor contains bacteria or viruses that prove to be pathogenic in the recipient – a different environment. Synthetic microbial communities should therefore substitute for donor faeces once they have been shown to be effective, e.g., in stimulating regulatory T cells or providing colonization resistance to *Salmonella* infection in the mouse (Narushima *et al*., 2014; Brugiroux *et al*., 2016). Judging pathogenic potential, however, is tricky because, as Casadevall and Pirofski (2014) have argued, there is no such thing as a ‘pathogen’ as the outcome of the interaction depends on the environment, for example, on the immune response of the host, which will vary according to the current physiological state of the host, its microbiota and diet. In other words, it is not just about the microbe but about the interactions in this complex system. Mathematical models that describe the activities of the ‘pathogen’ and other microbes and the host cells should be able to predict how they will interact. This could be used to evaluate under which physiological conditions of the host–microbiota holobiont a given microbe or set of microbes could cause disease and used to recommend that a particular patient should not be treated with a particular donor's or synthetic microbiota.

Mathematical models have been hugely successful in theoretical physics where predictions such as the existence of gravitational waves or particles like the Higgs boson are trusted to an extent that enormous efforts are funded to test these predictions. Theoretical biology has not yet had similar success, because biology is so diverse and complex and therefore more challenging to model and predict. Nevertheless, the ever‐increasing computational power, improvement of suitable tools for developing and simulating computational models and massive growth in available data will see this change over this century. The rise of mathematical approaches is now mainly hindered by the lack of a modelling tradition in biomedical and biological fields, where ‘model’ usually refers to animal or *in vitro* models, and mathematical models are ignored or met with scepticism despite a growing number of successful applications. For example, Cremer *et al*. (2017) have developed a mathematical model that provides insight into the microbiota interaction with pH and water flow in the gut. McLoughlin *et al*. (2016) have used models to examine how the host can favour beneficial over detrimental bacteria by differentially manipulating adhesion. Other successful examples come from biofilm modelling, which started in engineering – a field with a strong tradition of predictive modelling. Biofilm modelling has helped design a number of novel processes for wastewater treatment plants that have generated jobs in research‐driven engineering companies such as Paques B.V. in the Netherlands. Nadell *et al*. (2013) discuss mathematical approaches commonly used in bioengineering that would help biologists make sense of complex communities of microbes. We have argued that individual differences between cells should be considered in mathematical models to better represent the distribution of characteristics and to account for the effects of individuality on ecosystem function (Hellweger *et al*., 2016). In conclusion, microbial community research can replicate the success of physics if it takes mathematical modelling on board – it will be both challenging and rewarding (Widder *et al*., 2016).

## Conflict of interest

None declared.
